# Psoas Muscle Impingement by a Lumbar Disc-Osteophyte Complex: A Case Report

**DOI:** 10.7759/cureus.59790

**Published:** 2024-05-07

**Authors:** Yusuf Omran Hasan, Tariq Elkhalifa

**Affiliations:** 1 Trauma and Orthopaedics, Salmaniya Medical Complex, Manama, BHR

**Keywords:** spine, radiculopathy, disc-osteophyte, impingement, psoas

## Abstract

Psoas tendon impingement is not a frequently encountered condition, but impingement at the muscular level is not reported in the literature. The term refers to the mechanical impingement of the psoas muscle with secondary myositis. We report a case of psoas muscle impingement by a lumbar disc-osteophyte complex.

This study reports on a 61-year-old female who presented to our facility complaining of severe low back pain with increased intensity in the past two weeks. Radiographic imaging and magnetic resonance imaging revealed psoas muscle impingement by lumbar disc-osteophyte. No signs of nerve compression or infection were found. The patient responded well to conservative treatment, including non-steroidal anti-inflammatory drugs and physical therapy. No studies have reported psoas impingement syndrome due to the lumbar disc-osteophyte complex. More research is needed to better understand this condition.

## Introduction

Psoas tendon impingement is not a frequently encountered condition, but impingement at the muscular level is not reported in the literature. The term refers to the mechanical impingement of the psoas muscle with secondary myositis. Epidemiological data are not present as this is an uncommon finding; however, a visual vignette was published by LaBan et al. (2015) with similar radiological findings [1].

The psoas muscle is a primary flexor of the hip joint but its secondary rotational action is controversial [2]. It overlies the vertebral column on each side and combines with the iliacus muscle to form the iliopsoas muscle. The common tendon attaches to the lesser trochanter of the femur. Pathology of the psoas can present in various ways due to the proximity of the muscle to the internal organs, the relationship of the lumbar plexus and the femoral nerve, and the multiple fascial attachment [3].

## Case presentation

A 61-year-old female presented to our facility complaining of frequent severe low back pain with an intensity of 7/10 on a numeric rating scale (NRS) of 0-10, where 10 is maximum pain, with increased intensity for two weeks. She had been diagnosed two years prior with lumbar degenerative moderate canal stenosis at L4-L5. The patient reported that her pain was caused by movement and reduced by bending her hips and knees. Her past medical history included type 2 diabetes mellitus and a recent urinary tract infection.

An examination was performed, including palpation and range of motion (ROM), and a neurological exam revealed localized tenderness over the thoracolumbar spine junction with increased tenderness on the right. The neurological exam of the lower limb was unremarkable and there were no signs of radiculopathy. The patient was afebrile and her full blood count and serum electrolytes were unremarkable. The patient's​ white blood cell (WBC)​​ was 7.17 x 10^9 /L (the normal range of WBC is 4.5-11.0 x 10^9/L).

**Figure 1:**
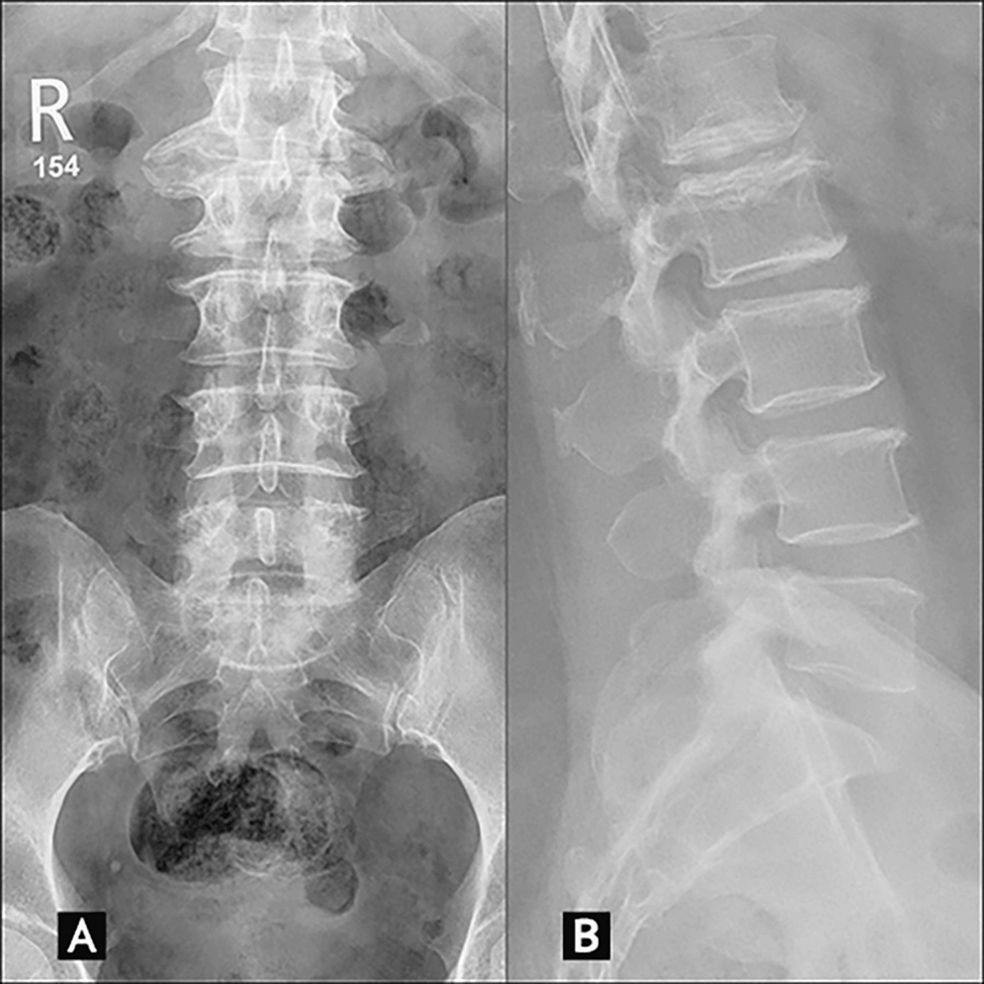
Lumbar X-ray shows at the level of L1-L2 (A) a large disc-osteophyte complex on the anteroposterior view and (B) sclerosis of endplates on the lateral view.

Lumbar radiographic examination of the patient revealed disc narrowing from T12-L4, bony sclerosis of endplates at T12-L5, and a large disc-osteophyte complex at L1-2, most evident on the anteroposterior (AP) lumbar radiograph (Figure 1).

**Figure 2:**
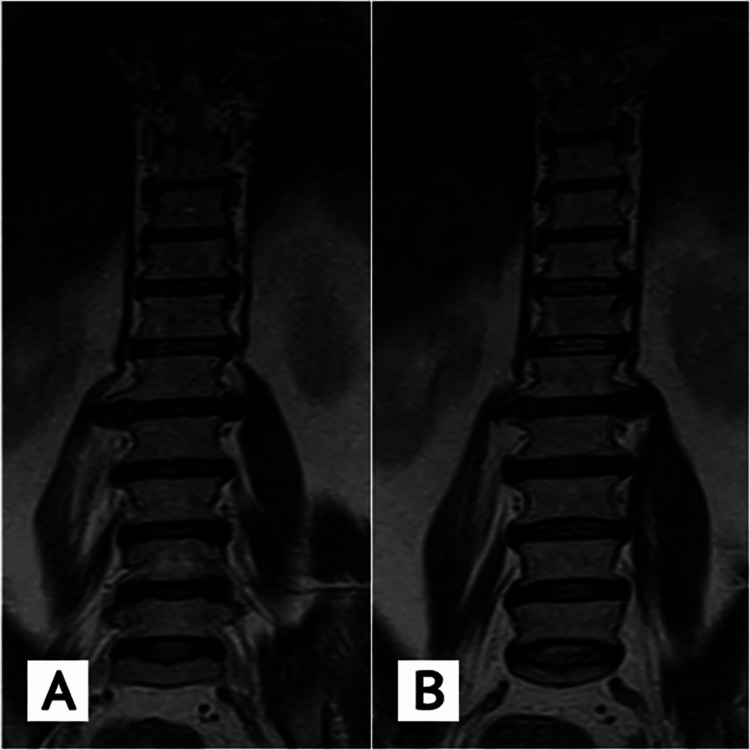
T2 coronal MRI images of the lumbosacral spine show (A & B) a large lateral disc osteophyte complex more on the right side indenting the right psoas muscle with an abnormal bright signal noted along the whole muscle.

Lumbosacral magnetic resonance imaging (MRI) of the patient revealed a large lateral disc-osteophyte complex, more prominent on the right side and indenting the right psoas muscle. There was an abnormal bright signal noted along the whole right psoas muscle, more prominent at the level of the L1-L2 disc protrusion. There was intense focal enhancement on post-contrast images predominantly focused around the protruded lateral disc at the L1-L2 level on the right side and less prominent enhancement on the left side (Figure 2).

There was no bright signal on the T2-weighted image or enhancement on the post-contrast image at the L1-L2 disc as well as no bone marrow edema ruling out signs of discitis.

**Figure 3:**
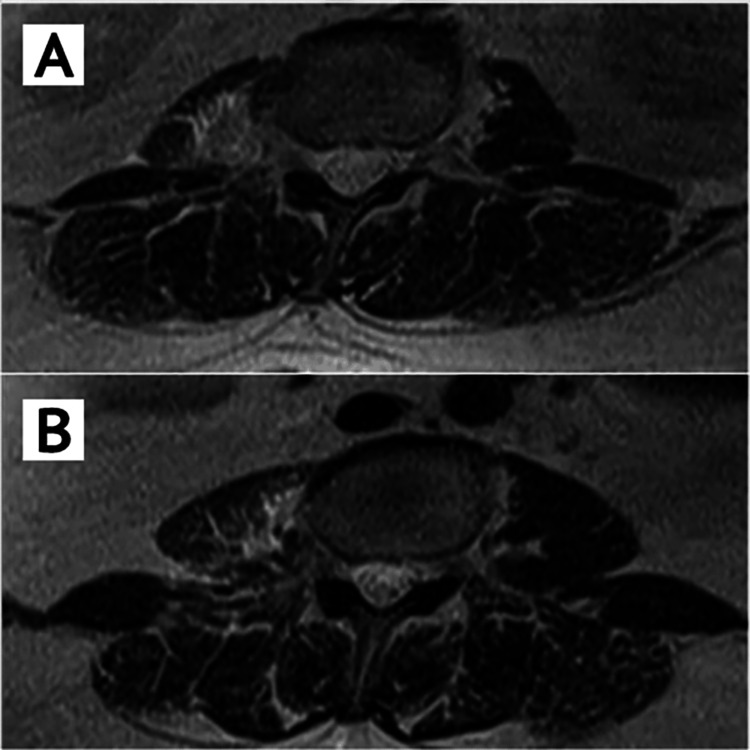
T2 axial MRI images (A) at L1-L2 level showing mild posterior disc herniation causing insignificant changes. No central canal stenosis or root compression (B) at the L2-L3 level was noted.

Mild posterior disc herniation was seen at L1-L2 on the axial images with no central canal stenosis (Figure 3). The L1-L2 lateral disc-osteophyte protrusion did not create any extra exiting nerve root compression in the L1-L2 foramina.

Based on the advanced imaging, it was determined that the large disc-osteophyte complex at L1-L2 caused the right psoas muscle impingement. The patient responded well to conservative treatment. She was prescribed non-steroidal anti-inflammatory drugs (NSAIDs) for four weeks and physical therapy, including functional, manual, and electric stimulation physiotherapy. At a one-year follow-up exam, the patient reported that her lower back pain was infrequent at 2/10 on the NRS. She was discharged from care.

## Discussion

Psoas muscle impingement by lumbar disc-osteophyte complex is extremely rare in published literature. A search in the literature included MEDLINE and Embase. The search produced one visual vignette case report of mechanical compression of the iliopsoas muscle by a large vertebral osteophyte. In the present case, the clinical findings from physical examination and MRI confirmed the diagnosis. That said, a differential diagnosis including nerve root compression, spondylodiscitis, and Charcot’s spine should be considered and ruled out first.

Nerve root compression commonly presents as posterolateral radiating pain down the lower extremity and may be accompanied by paresthesia or weakness. It is caused by any mass compressing the nerve root, commonly by disc herniation. MRI findings are variable ranging from disc protrusion to a completely extruded disc, which can be central, foraminal, or extraforaminal at the pedicle level or the levels above or below. Careful evaluation in three planes is essential when making this diagnosis or when surgical intervention is planned [4].

Spondylodiscitis is a serious condition associated with high morbidity and mortality rates. Patients over 50 years of age, taking steroids, who had a recent urinary tract infection (UTI) or surgery, or those with type 2 diabetes mellitus, human immunodeficiency virus (HIV), or rheumatoid arthritis (RA) are at increased risk of developing spinal infection [5]. In adults, vertebral endplates get affected first, followed by the joint space, then the vertebral body, and finally the surrounding tissue. MRI typically shows destruction of the endplate, marrow edema, abscess collection, and extension to the paravertebral space, psoas muscle, or epidural space causing compression.

Charcot spine arthropathy (CSA) is less common than Charcot arthropathy of the lower limbs. Unlike degenerative arthropathy, mobility is preserved and proprioception is lost [6]. This leads to instability, increased stress on the spinal functional unit, and joint subluxation or dislocation. In an attempt to increase stability, exuberant, nonbridging peripheral callus forms. Patients usually complain of localized back pain rather than radiculopathy. Treatment with fusion has been shown to be successful in stabilizing the spine and reducing CSA symptoms [6].

## Conclusions

Psoas muscle impingement by lumbar disc-osteophyte complex is extremely rare in published literature. Only one other visual vignette case report of mechanical compression of the iliopsoas muscle by a large vertebral osteophyte exists. Psoas muscle impingement by lumbar disc-osteophyte complex should be a diagnostic consideration for back pain triggered by hip movement with no neurological findings and radiographic or advanced imaging suggestive of this condition.
